# A study on the correlations between parent–child interaction, school adaptation type, and adolescent mental health

**DOI:** 10.3389/fpsyg.2026.1734974

**Published:** 2026-08-27

**Authors:** Xinqian Yin, Yan Wang, Yashan Qu, Lanlan Luo

**Affiliations:** 1 School of Marxism, Guilin Medical University, Guilin, China; 2 School of Education, Guangxi Normal University, Guilin, China; 3 Key Research Base of Humanities and Social Sciences in Guangxi Universities Guangxi Higher Education Development Research Center, Guilin, China; 4 School of Humanities and Management, Guilin Medical University, Guilin, China; 5 School of Educational Sciences, Guangdong Polytechnic Normal University, Guangzhou, China

**Keywords:** adolescents, depressive symptoms, happiness index, mental health, parent–child interaction, school adjustment

## Abstract

**Introduction:**

This study aimed to explore the associations between different parent–child interaction patterns, school adjustment types, and multidimensional mental health indicators such as adolescent wellbeing indices and depressive symptoms and thereby provide insights for improving adolescent mental health.

**Methods:**

Using questionnaire data from the China Education Panel Survey (CEPS) conducted from spring 2014 to spring 2015, this study focused on school-attending adolescents aged 12–18 years. Data from the Parent–Child Interaction Questionnaire, the School Adjustment Questionnaire, and the Mental Health Questionnaire were used. Cluster analysis using the K-means algorithm, combined with non-parametric tests and the analysis of variance (ANOVA) with multiple comparisons, identified distinct parent–child interaction patterns and school adjustment types and examined their associations with adolescent wellbeing indices and depressive symptoms.

**Results:**

Depressive symptoms showed negative correlations with positive school adaptation, parent–child interaction frequency, and parent–child interaction content (*r* = −0.247, −0.123, −0.16, *p* < 0.001), while wellbeing exhibited positive correlations (*r* = 0.304, 0.135, 0.123, *p* < 0.001). Parental interaction patterns were categorized into high-frequency (35%), medium-frequency (38%), and low-frequency (27%) groups. School adaptation types were classified as high-involvement adaptation (50%), balanced adaptation (35.3%), and alienated adaptation (15%). Different parent–child interaction patterns significantly influenced adolescents’ mental health (depression: *F* = 743.821, *p* < 0.001, ηp^2^ = 0.083; wellbeing: *F* = 795.63, *p* < 0.001, ηp^2^ = 0.079). Different school adaptation types also significantly influenced adolescents’ mental health (depression: *F* = 135.777, *p* < 0.001, ηp^2^ = 0.022; wellbeing: *F* = 190.848, *p* < 0.001, ηp^2^ = 0.016). Multiple comparison analyses revealed significant differences between different parent–child interaction patterns and school adaptation factors in their prediction of adolescent mental health outcomes. The predictive effect on the wellbeing index was ΔR^2^ = 0.087 (*F* = 761.6, *p* < 0.001), while the predictive effect on depressive symptoms was ΔR^2^ = 0.064 (*F* = 529.3, p < 0.001).

**Conclusion:**

The combination of high-frequency parent–child interaction and high-investment school adaptation is most conducive to enhancing adolescents’ wellbeing and alleviating depressive symptoms. This finding provides theoretical guidance and empirical evidence for interventions addressing adolescent mental health issues.

## 1 Introduction

Adolescence is a peak period for individual physical and mental health development and serves as a transitional phase from the family to the broader society, holding significant importance in human socialization processes (Jiang et al., 2025). Mental health issues among adolescents have become a major public health concern (Tinsae et al., 2024). According to the World Health Organization statistics (World Health Organization, 2024), globally, one in seven 10–19-year-olds experiences a mental disorder, accounting for 15% of the global burden of disease in this age group (2024). In recent years, the suicide rate among adolescents in China has shown a continuous upward trend, and mental health issues such as depression and excessive anxiety have become increasingly prominent.

The mental health of adolescents is influenced by multiple factors, including stress, insomnia, and fatigue (Bångstad et al., 2022); problematic internet use (Dadi et al., 2024); physical activity (PA) and family economic status (Wang H. et al., 2025); and the school environment (Neill et al., 2025). Among these, the home environment and school environment are important areas for intervention. Research indicates that parent–child interaction and school adjustment are core environmental factors influencing adolescent mental health (Sabah and Alduais, 2025; Xie et al., 2022). From a family perspective, the quality of parent–child interaction directly shapes adolescents’ psychological capital and behavioral patterns (Zhou et al., 2025; Kim and Carlin, 2025). High-quality parent–child relationships can significantly enhance adolescents’ self-esteem and self-control, thereby effectively reducing psychological risks such as smartphone dependence (Li et al., 2025). This indicates that parent–child interaction exerts a profound influence on adolescent development through the mediating role of psychological mechanisms (Wang et al., 2024). At the same time, a positive self-image and good communication skills often stem from healthy parent–child interactions, and the two exhibit a significant positive correlation (Bilukha et al., 2024). Positive parent–child interactions (such as supportive communication) significantly promote adolescents’ prosocial behavior and positive emotions (Man et al., 2022), while negative interaction patterns (such as authoritarian parenting (Seppälä et al., 2024) or parental alienation after divorce (Reiter et al., 2013; Miralles et al., 2023)) exacerbate self-negativity and emotional disorders. High-frequency parent–child interaction can mitigate adolescents’ trauma from abuse (Shan et al., 2019; Griffith et al., 2025), while low-frequency interaction increases their risk of depression (Twork et al., 2025).

It is worth noting that the influence of the family system is not limited to within the family but also exhibits a significant “cross-context generalization effect” (Bronfenbrenner and Morris, 2006; Rothenberg et al., 2023). Other studies have shown that improving the quality of parent–child interactions can effectively enhance children’s compliance and adaptability at school, demonstrating that positive interventions at the family level can generate spillover effects that promote positive adaptation at the school level (McNeil et al., 1991; Wang and Liu, 2025). However, the interactive effects between the family and school systems are complex, and not all types of family involvement or interaction are beneficial for school adaptation (Tang et al., 2025). Research has found that direct parental involvement at the school level may sometimes be associated with lower academic performance or school satisfaction among children, whereas interpersonal interactions within the family (such as communication and companionship) have a more consistent positive predictive effect on psychological adjustment (Tan and Goldberg, 2009; Liu et al., 2025). This suggests that we need to distinguish between different patterns of parent–child interaction and types of school adjustment to accurately understand their impact pathways.

At the school level, good adaptation reduces the occurrence of adolescent problem behaviors and dropout (Kupersmidt and Coie, 1990; Xie and Fang, 2026), whereas maladaptation leads to academic failure, behavioral issues, and poor social adjustment (Mauguan, 1994; Azpiazu et al., 2025). Concurrently, multiple pressures—high academic expectations, complex peer relationships, and family demands—can cause adolescents to experience emotional dysregulation, sleep disorders, and impaired learning efficacy (Zhou et al., 2023). Existing research provides a foundation for demonstrating the impact of parent–child interaction and school adjustment on adolescent mental health. However, a majority of the studies focus on the singular effect of either parent–child interaction (Hou et al., 2025) or school adjustment (Hei et al., 2025) on mental health, lacking in-depth exploration of the associations between different parent–child interaction patterns, different types of school adjustment, and adolescent mental health. Furthermore, a majority of the studies inadequately address multidimensional mental health outcomes (Sabah et al., 2025; Zeng et al., 2026), with few studies systematically examining their relationships with both positive psychological states (e.g., wellbeing indices) and negative psychological symptoms (e.g., depressive symptoms) in adolescents. Therefore, based on cross-sectional data from the China Education Panel Survey (CEPS), this study aims to explore the associations between different parent–child interaction patterns and school adjustment types with multidimensional mental health indicators (wellbeing index and depressive symptoms) among adolescents. It seeks to provide new perspectives and theoretical foundations to improve adolescents’ wellbeing and reduce depressive symptoms.

It should be noted that, although the CEPS data were collected in 2014–2015, they remain the most recent nationwide longitudinal survey data of adolescents currently available in China, they remain valid for interpretive purposes today and can serve as a reliable empirical reference for research on the relationship between parent–child interaction and school adjustment, as well as for understanding issues related to the mental health of Chinese adolescents. This is because Chinese parenting philosophies and parent–child interaction patterns are deeply influenced by Confucian ethics and culture (Guo et al., 2026), and these relationship patterns and parenting behaviors exhibit deep-rooted cultural inertia (Cui, 2023). Furthermore, China’s basic education system (Xie et al., 2025), the college and high school entrance examination-centered selection mechanism (Zhang and Ma, 2026), and the home-school cooperation system have remained relatively stable (Guo et al., 2025). Consequently, institutional structures and sources of pressure affecting adolescents’ school adjustment have not undergone fundamental changes.

Specifically, this study examined the following questions and proposed four hypotheses:

*H1*: Parent–child interaction and school adjustment are significantly correlated with mental health indicators;

*H2*: Different patterns of parent–child interaction have a significant impact on adolescents’ mental health;

*H3*: There are significant differences in mental health levels among adolescents with different types of school adjustment;

*H4*: Parent–child interaction patterns and school adjustment types have a significant joint predictive effect on adolescents’ mental health.

## 2 Methods

### 2.1 Participants

This study focused on school-aged adolescents aged 12–18 in China, using data from the China Education Panel Survey (CEPS) funded by the China Survey and Data Center at Renmin University of China. Data collection spanned from spring 2014 to spring 2015, targeting seventh-grade junior high and ninth-grade senior high students. The survey used stratified, multistage, and probability-proportional-to-size (PPS) sampling. Using a school-based sampling frame, a nationally representative sample of 20,000 students was selected from 112 schools and 438 classes across 28 county-level administrative regions nationwide. See Table 1.

**Table 1:** Sample demographics of participants.

Variable	*N*	*M (SD)*	range	χ^2^	*P*
Age	19,082	1999.47 (1.24)	1996–2002	11,286.96	0.000
Gender	19,217	1.48 (0.5)	1–2	14.89	0.000
Nation (minority)	1,943 (1,697)	1.51 (1.76)	1–8	94,501.94	0.000
Socioeconomic position	18,594	1.66 (0.797)	1–4	10,980.75	0.000

It should be noted that the CEPS baseline survey was designed to follow two specific grade cohorts: seventh-grade (first-year junior high) and ninth-grade (third-year junior high) students. The CEPS applied a stratified, multistage sampling design with probability proportional to size (PPS), randomly selecting a school-based, nationally representative sample of approximately 20,000 students from 438 classrooms of 112 schools in 28 county-level units in China (CEPS, 2014). The restriction to these two grade levels was determined by the original survey design rather than by the present researchers’ selection criteria. Consequently, other grade levels within the 12–18 age range (e.g., eighth-grade junior high students or senior high school students) were not available in this dataset. While this may limit the generalizability of findings to the full adolescent age spectrum, the CEPS sample remains nationally representative for the two targeted grade cohorts, and prior research has demonstrated that this dataset provides reliable estimates of adolescent developmental outcomes in China (Li et al., 2022; Li L. et al., 2024). The inclusion criteria for the present study were as follows: (a) valid responses to the parent–child interaction questionnaire, (b) valid responses to the school adaptation questionnaire, and (c) complete data on at least one mental health outcome measure. Cases with missing data on all key variables were excluded.

### 2.2 Methodology

The CEPS questionnaires assess children’s school life, mental health status, and parent–child interactions. This survey used five distinct instruments administered to students, parents, homeroom teachers, subject teachers, and school administrators. The questionnaires covered local education policies, the school curriculum structure, teacher-student relationships, peer relationships, family environment, family education processes, parent–child relationships, and home-school relationships. This study used data from three of these questionnaires; the variables involved are detailed in Table 2.

**Table 2:** Variable definitions, sub-dimensions, indicator items, and response scales.

Variable	Dimension	Items	Value description
Parent -child interaction	Interaction frequency	Over the past year, families have engaged in the following activities with their children: dinner / reading / watching TV / doing sports / visiting museums, zoos, science museums, etc. / going out to see performances, sports events or movies	1 = Never done; 2 = once a year; 3 = every 6 months; 4 = once a month; 5 = once a week; 6 = once a week or more
	Interaction content	Do you take the initiative to discuss with your child: what is happening at school / the relationship between your child and his friends / the relationship between your child and his teachers / how your child feels / what is on your child’s mind or worries	1 = Not true; 2 = Somewhat true; 3 = Certainly true
School adaptation	Active adaptation	About school life, do you agree with the following statements: The head teacher often praises me / most of my classmates are friendly to me / I think I get along well with others / the class atmosphere in my class is good / I often participate in activities organized by the school or class / I feel close to people in this school	1 = Definitely does not apply; 3 = Neutral/not sure; 5 = Definitely applies
	Negative adaptation	About school life, do you agree with the following statements: I am often late / I often skip classes / My parents often receive criticism from teachers / The head teacher often criticizes me / I feel bored in this school / I hope to go to another school	1 = Definitely does not apply; 3 = Neutral/not sure; 5 = Definitely applies
Mental health	Wellbeing Index	Even if I’m a little sick or have other reasons to stay home, I still try to go to school / Even if I do not like the work, I will try my best to do it / Even if the homework takes a long time to finish, I will continue to try my best / I can express my opinions clearly / I am quick to react / I can learn new things quickly / I am curious about new things	1 = Definitely does not apply; 3 = Neutral/not sure; 5 = Definitely applies
	Depressive symptoms	Have you felt any of the following in the last 7 days: depressed / sad / unhappy / life is not interesting / sad	1 = Definitely does not apply; 3 = Neutral/not sure; 5 = Definitely applies

#### 2.2.1 Parent–child interaction questionnaire

The Parent–Child Interaction Questionnaire, completed by parents, measured two dimensions: interaction frequency and content. Previous research has confirmed that this scale possesses satisfactory psychometric properties (Wang and Zhao, 2022), making it a relatively mature measurement tool. In this study, the Cronbach’s alpha coefficients for the two dimensions were 0.766 and 0.866, respectively, indicating good reliability.

#### 2.2.2 School adjustment questionnaire

The School Adjustment Questionnaire, completed by parents, measures two dimensions: active adjustment and passive adjustment. Previous studies have confirmed that this scale possesses satisfactory psychometric properties (Zhou et al., 2024), making it a relatively mature measurement tool. In this study, Cronbach’s alpha coefficients for the two dimensions were 0.721 and 0.624, respectively, indicating good reliability.

#### 2.2.3 Mental health questionnaire

Mental health status was assessed using adolescent self-reported questionnaires and was evaluated using two indicators: the Wellbeing Index and the Depression Scale. The Wellbeing Index used the WHO-5 Wellbeing Index developed by the WHO Collaborating Centre for Mental Health Research. The Depression Scale was based on the Center for Epidemiologic Studies Depression Scale (CES-D) short form developed by the US National Institute of Mental Health (Li et al., 2022). Both scales are relatively well-established measurement tools (Zeng and Hong, 2020; Doumit et al., 2025), focusing on the positive and negative dimensions of mental health, respectively. Within theories of happiness and wellbeing, these dimensions correspond to the cognitive and affective components of mental health. In this study, the Cronbach’s alpha coefficients for the two scales were 0.782 and 0.859, respectively, indicating good reliability.

### 2.3 Statistical analysis

To address the research questions, we used a person-centered analytical approach, namely cluster analysis. This method identifies groups of children exhibiting similar response patterns across multiple target variables by examining the combined characteristics of individual samples across various dimensions (Magnusson and Bergman, 1988; Zhang et al., 2024). The K-means algorithm is suitable for clustering large samples (Oti et al., 2021; Peral-Suárez et al., 2024). This study randomly selected 3–5 cluster centers and iterated the K-means algorithm until convergence. Subsequently, the System Clustering algorithm calculated distances between data points to determine the optimal number of clusters. The chi-square tests, supplemented with standardized residuals, were then used to validate significant gender differences across clusters. The optimal clustering scheme was determined by examining the weighted class square sum (WCSS), pseudo-F statistic, and pseudo-T^2^ statistic (Milligan and Cooper, 1985; Shaikh et al., 2024). Lower WCSS values and higher pseudo-F and pseudo-T^2^ statistics indicate superior clustering performance. The final stage of cluster analysis involved defining and interpreting category characteristics. We described and delineated the metric features of each category using mean comparison methods. Finally, we used the analysis of variance (ANOVA) combined with Tukey’s *post-hoc* test to compare each outcome variable with the generated clusters. This approach both assessed the amount of variance explained by the clusters and examined specific differences in outcome variables across clusters. For missing data, we utilized the expectation–maximization (EM) algorithm for imputation. This method performs maximum likelihood estimation for unknown parameters, is suitable for large-sample studies, and effectively addresses missing data issues (Sammut and Webb, 2011; Lachos et al., 2025).

## 3 Results

### 3.1 Descriptive statistics

The means, standard deviations, and correlation coefficients for each variable are shown in Table 3. A majority of the correlation coefficients ranged from small to moderate. Depressive symptoms showed negative correlations with active adaptation, frequency of parent–child interaction, and content of parent–child interaction but positive correlations with negative adaptation. Conversely, the happiness index demonstrated positive correlations with active adaptation, frequency of parent–child interaction, and content of parent–child interaction, while exhibiting negative correlations with negative adaptation.

**Table 3:** Means, standard deviations, and correlations among variables.

Variable	Active adaptation	Negative adaptation	Interaction frequency	Interaction content	Depressive symptoms	Wellbeing index
Active adaptation	1					
Negative adaptation	−0.084**	1				
Interaction frequency	0.229**	−0.155**	1			
Interaction content	0.230**	−0.125**	0.450**	1		
Depressive symptoms	−0.247**	0.298**	−0.123**	−0.160**	1	
Well-being Index	0.304**	−0.186**	0.135**	0.123**	−0.177**	1
*N*	18,694	18,993	18,403	17,407	18,794	18,270
*SD*	0.03	0.02	0.02	0.05	0.03	0.27

**Significant correlation at the 0.01 level (two-tailed).

### 3.2 Cluster analysis

The K-means clustering algorithm was used to partition the samples into distinct groups. Before analysis, all variables were standardized using z-score transformation to eliminate differences in measurement scales. The optimal number of clusters was determined by combining the elbow method with the contour coefficient, with results shown in Table 4. Based on the criteria that a smaller WCSS is preferable and a larger pseudo-F statistic is desirable, both *K* = 3 and *K* = 4 met the fundamental requirements. Further analysis using the systematic clustering algorithm revealed that merging from 3 to 2 clusters increased the merging distance from 399,142 to 849,772, with a 112.9% increase. Merging from 2 to 1 clusters further increased the merging distance to 2,510,825, a 195.4% increase. Despite the larger leap in the final merger, the 3-cluster scheme offers more granular variable grouping. Calculating the pseudo-*T*^2^ statistic (pseudo-*T*^2^ = 239.4 ≥ 3 when merging 3 clusters into 2) further confirms extremely significant differences between clusters, indicating low merger rationality. The 3-cluster solution offers the best interpretability, clearly defining groups with distinct characteristics. The 2-cluster solution is overly broad, while the 4-cluster solution may be redundant. Further stability validation using bootstrapping (500 bootstrap iterations) revealed that the *K* = 3 scheme achieved a Class Membership Consistency Index (ACI) of 0.89. Concurrently, the contour coefficient was 0.61 (>0.5), indicating a robust clustering structure. Therefore, considering both statistical metrics and theoretical interpretability, the *K* = 3 scheme was achieved as the optimal choice.

**Table 4:** Comparison of K-means clustering fit indices (*K* = 2–5).

Number of clusters	Variable	WCSS	Pseudo-F	Minimum cluster proportion
*K = 2*	School adaptation	335,468.61	11,780.86	>5%
	Parent–child interaction	300,907.96		
*K = 3*	School adaptation	322,865.6	8,577.60	>5%
	Parent–child interaction	211,282.63		
*K = 4*	School adaptation	228,573.68	7,533.00	>5%
	Parent–child interaction	230,828.84		
*K = 5*	School adaptation	207,060.05	6,615.00	>5%
	Parent–child interaction	210,947.55		

Regarding parent–child interaction, the total sample (*n* = 17,110) was classified into three clusters: the high-frequency interaction group, characterized by rich interaction content and the highest interaction frequency (*n* = 5,963, 35%); the medium-frequency interaction group, featuring general interaction content and moderate interaction frequency (*n* = 6,490, 38%); and the low-frequency interaction group, characterized by limited interaction content and the lowest frequency (*n* = 4,657, 27%).

Regarding school adaptation, the total sample (*n* = 18,403) was classified into the three-cluster scheme: the high-participation adaptation group, with the highest positive adaptation levels and lowest negative adaptation levels (*n* = 9,272, 50%); the balanced adaptation group, with low levels of both positive and negative adaptation (*n* = 6,371, 35%); and the detached adaptation group, with moderate levels of both positive and negative adaptation (*n* = 2,832, 15%). Table 4 presents the standardized means for key variables across groups.

Significant between-group differences were observed for all variables (*p <* 0.01), confirming discriminant validity (F statistic and *p* values in Table 4). Regarding parent–child interactions, chi-square tests revealed significant associations between cluster groups and demographic variables (country: χ^2^ = 593.337, *p <* 0.01; age: χ^2^ = 703.322, *p <* 0.01), indicating distinct demographic differences across clusters. Regarding school adjustment, the chi-square test revealed significant associations between cluster groups and demographic variables (gender: χ^2^ = 138.079, *p <* 0.01; ethnicity: χ^2^ = 269.693, *p <* 0.01; age: χ^2^ = 264.422, *p <* 0.01), similarly indicating substantial demographic differences across distinct cluster groups (Table 5).

**Table 5:** Cluster center means (standard deviations) and discriminant validity tests for parent–child interaction and school adjustment.

Variable	Class 1	Class 2	Class 3	R^2^	*F*
Interaction frequency	12.79 (0.027)	11.51 (0.027)	9.464 (2.52)	0.242	2,816.071**
Interaction content	28.88 (0.038)	22.04 (0.026)	14.53 (0.042)	0.817	38,468.051**
Positive adaptation	21.86 (0.021)	15.18 (0.0345)	19.93 (0.051)	0.179	14,285.439**
Negative adaptation	7.317 (0.013)	8.39 (0.024)	13.02 (0.051)	0.442	10,386.601**

**Significant at the 0.01 level (two-tailed). F, F statistic; R^2^, coefficient of determination.

### 3.3 Analysis of variance

Since the data did not satisfy the assumption of homogeneity of variance (Levene’s test, *p <* 0.05), this study used non-parametric tests and multiple comparison methods to examine the main effects and interaction patterns of parent–child interaction styles and school adaptation patterns on adolescent depressive symptoms and wellbeing indices. Table 6 presents the ANOVA results for each outcome measure. Although all comparisons reached statistical significance, the eta-squared values indicated small effect sizes: *ηp^2^* values for wellbeing indices and depressive symptoms were 0.079/0.016 and 0.083/0.022, respectively.

**Table 6:** Analysis of variance results for parental interaction and school adjustment types on mental health.

Variable		Class 1	Class 2	Class 3	*F*	*η^2^*	*Post hoc* comparison
Happiness index	a	23.55 (0.037)	21.38 (0.027)	21.64 (0.071)	743.821**	0.079	1 > 3 > 2
	b	23.11 (0.052)	22.57 (0.045)	21.89 (0.053)	135.777**	0.016	1 > 2 > 3
Depressive symptoms	a	9.28 (0.037)	11.36 (0.053)	12.11 (0.08)	795.63**	0.083	3 > 2 > 1
	b	9.70 (0.053)	10.37 (0.050)	11.27 (0.02)	190.848**	0.022	3 > 2 > 1

**Significant at the 0.01 level (two-tailed); a = parent–child interaction style; b = school adaptation style.

The results demonstrated that different parent–child interaction patterns exerted statistically significant effects on depressive symptoms (*F =* 743.821, *p <* 0.001) and wellbeing index (*F =* 795.63, *p <* 0.001). Different school adaptation patterns also significantly influenced depressive symptoms (*F =* 135.777, *p <* 0.001) and wellbeing index (*F =* 190.848, *p <* 0.001). Multiple comparison analyses further revealed significant differential effects between different parent–child interaction patterns and school adaptation factors in predicting health outcomes (wellbeing index: *ΔR^2^* = 0.087, *F =* 761.6, *p <* 0.001; depressive symptoms: *ΔR^2^* = 0.064, *F =* 529.3, *p <* 0.001).

Effect sizes were interpreted according to Cohen’s (1988) guidelines for partial eta-squared (*η^2^*): values of 0.01, 0.06, and 0.14 represent small, medium, and large effects, respectively (Cohen, 1988). The effect size for parent–child interaction on depressive symptoms (*η^2^* = 0.083) was medium, indicating that the parent–child interaction patterns explained approximately 8.3% of the variance in depressive symptoms. The effect of school adaptation on depressive symptoms (*η^2^* = 0.022) was small, explaining approximately 2.2% of the variance. For wellbeing, the effect of parent–child interaction (*η^2^* = 0.079) was medium, explaining approximately 7.9% of the variance, while the effect of school adaptation (*η^2^* = 0.016) was small, explaining approximately 1.6% of the variance. Overall, the parent–child interaction patterns showed medium-sized effects on both mental health indicators, whereas school adaptation patterns showed small effects. The wellbeing index results showed that the high-frequency parent–child interaction with high-investment school adaptation group scored significantly higher than the low-frequency parent–child interaction with balanced school adaptation group, as well as the medium-frequency parent–child interaction with the detached school adaptation group. Depressive symptom data indicated that the low-frequency parent–child interaction with the detached school adaptation group scored higher than the medium-frequency parent–child interaction with the balanced school adaptation group and significantly higher than the high-frequency parent–child interaction with the high-investment school adaptation group. This indicates that high-frequency parent–child interaction combined with a high-engagement school adaptation pattern is most conducive to enhancing adolescents’ happiness index and alleviating depressive symptoms.

## 4 Discussion

This study, using cross-sectional data, reveals significant associations between the frequency and content of parent–child interactions and adolescents’ depressive symptoms and wellbeing indices. High-frequency parent–child interactions combined with a high-engagement school adaptation approach were most beneficial for improving depressive symptoms and enhancing wellbeing development in adolescents.

### 4.1 Parent–child interaction patterns and adolescent mental health

The findings indicate that adolescents’ parent–child interactions can be categorized into three patterns: high-frequency, high-quality interactions; medium-frequency, medium-quality interactions; and low-frequency, low-quality interactions. Previous research indicates that parent–child interaction plays a crucial role in child development, with interaction frequency showing significant associations with children’s physical and mental health (Twork et al., 2025). Parent–child interaction encompasses three dimensions: frequency, content, and style (Li, 2023). Research indicates that harmonious parent–child relationships can reduce the probability of adolescents being in troubled and vulnerable states and promote their transition from troubled and vulnerable to complete mental health (Wang Y. et al., 2025; Wang H. et al., 2025; Wang X. et al., 2025). However, unlike previous studies focusing solely on positive (Shee et al., 2025) and negative interaction patterns (Chen et al., 2024), this research highlights the previously understudied mid-frequency interaction group (Lee and Wong, 2022). This finding indicates that not all parent–child interaction frequencies exist at extremes; an intermediate level exists. Data from American adolescents indicate that the low-frequency interaction group accounts for up to 28.6% of the population and is strongly correlated with academic adjustment issues (Lee and Wong, 2022). This contrasts with the findings of this study, in which the proportion of Chinese adolescents in the low-frequency interaction group is 17.3%. This discrepancy may be related to the higher expectations and close bonds inherent in parent–child relationships within the Chinese cultural context, as well as the prevalent notion of “academic supremacy” (Pasupathi et al., 2022). More importantly, this study found that the parent–child interaction patterns explained mental health indicators more effectively than school adaptation patterns, highlighting the core role of the family environment.

From a theoretical perspective, these findings can be understood through family systems theory (Cox and Paley, 1997; Miller and Elder, 2025), which posits that the family interaction patterns create relational contexts that shape members’ psychological functioning. High-frequency, high-quality parent–child interactions reflect a well-functioning family system characterized by open communication, emotional support, and clear boundaries. Such interactions likely fulfill adolescents’ basic psychological needs for relatedness and autonomy, as described by the Self-Determination Theory (Ryan and Deci, 2017; Núñez-Regueiro et al., 2024), thereby promoting psychological wellbeing and buffering against depressive symptoms. Therefore, intervention strategies for adolescent mental health should prioritize parent–child interaction, particularly targeting groups with low-frequency, low-quality interactions. Community or school-organized parent–child training courses can guide parents in mastering effective communication skills and interaction methods, focusing on enhancing the frequency and quality of interactions for adolescents in low-frequency parent–child interaction groups.

### 4.2 School adaptation types and adolescent mental health

The findings indicate that adolescents’ school adaptation manifests in three patterns: high adaptation, low adaptation, and conflict adaptation. School adaptation is fundamentally a dynamic process of individual-school environment interaction. Strengthening teacher support and fostering students’ self-esteem are effective strategies for improving school adjustment (Jiang et al., 2026). Research indicates diversity in adolescent school adaptation patterns (Leonard and Gudiño, 2021; Yang et al., 2025). Unlike previous studies focusing on single dimensions such as academic performance (Xu et al., 2025), teacher–student relationships (Fang et al., 2025), or peer interactions (Lu et al., 2024) to assess adolescent mental health, our findings reveal that the core dimensions of positive adaptation and negative adaptation effectively distinguish distinct adaptation patterns. Notably, the “conflicted adaptation group” (15% of participants) identified in this study exhibits high positive adaptation alongside the highest negative adaptation. This unique “high investment, high stress” state is associated with the lowest wellbeing index and highest depressive symptoms. Additionally, the low adaptation group exhibits the lowest levels of positive adaptation in emotional, behavioral, and cognitive domains, coupled with moderately high negative adaptation levels. Their wellbeing index and depressive symptoms fall between those of the high adaptation group and the conflict adaptation group. School interventions should therefore be more targeted: For the conflict adaptation group, the focus should be on identifying and alleviating stressors, optimizing psychological load management under high-investment conditions, and preventing high consumption resulting from high investment. For the low adaptation group, campus activities should be diversified, incorporating varied cultural, artistic, and athletic programs. Personalized guidance and support should be provided to students with low participation rates to enhance adolescent school adaptation.

These findings align with Bronfenbrenner’s (1979) bioecological model, in which school represents a critical microsystem shaping adolescent development (Bronfenbrenner, 1979; Tong and An, 2024). Within this framework, school adaptation reflects the quality of person-environment fit: The high adaptation group achieves congruence between their needs and the school environment, while the conflicted adaptation group experiences a mismatch, with high behavioral investment accompanied by excessive psychological demands. Self-Determination Theory further explains this pattern: The conflicted group may satisfy their need for competence through active participation; however, they may simultaneously experience thwarted autonomy due to perceived pressure, resulting in the paradoxical combination of high engagement and poor wellbeing (Ryan and Deci, 2017; Zhang and Jiang, 2023). This theoretical lens suggests that interventions for the conflicted adaptation group should focus on restoring a sense of autonomy and reducing perceived coercion rather than simply encouraging more engagement.

### 4.3 The combined effects of parent–child interaction and school adaptation on mental health

This study further reveals that high-frequency, high-quality parent–child interaction patterns combined with highly adaptive school environments most effectively enhance adolescent wellbeing and alleviate depressive symptoms. Communication between parents and adolescents can influence adolescents’ school experiences and academic difficulties, which, in turn, can affect their depressive symptoms (Wang et al., 2022). Moderate-frequency interaction may represent the optimal balance between parental autonomy support and behavioral regulation and may play a crucial role in adolescent school adaptation (Zhou et al., 2025). Peer attachment mediated the association between parental relationships and depressive symptoms, aligning with our finding that the family interaction quality associates with depression levels. Parent–child discrepancies in educational aspirations and their associations with Chinese adolescents’ school adaptation, reporting that congruent parent–child aspirations are linked to better school adaptation, providing additional evidence for the interconnection between family dynamics and school adjustment in our study (Sun et al., 2025). Notably, our study indicates that, within the Chinese cultural context, high-frequency, high-quality parent–child interaction provides adolescents with abundant emotional support and positive behavioral guidance, exerting a more significant promotional effect on their mental health. This aligns with traditional Confucian filial piety and parenting concepts that emphasize strong parental concern for offspring (Kim et al., 2025). Furthermore, supportive parenting and meeting basic psychological needs (Abidin et al., 2022), along with the moderating effects of parental educational aspirations and physical health disparities (Zeng et al., 2024), facilitate school adjustments to enhance adolescents’ adaptation to school life. Research indicates that schools play a significant role in influencing health and education outcomes. Teachers’ cultural beliefs effectively translate into students’ school adaptation (Schotte et al., 2022). The formation of a highly adaptive school model may depend on a multidimensional support system (Fang et al., 2025). Secure peer attachment and good social support jointly buffer against depressive symptoms in adolescents (Sun et al., 2025). Specifically, teachers can indirectly promote school adaptation by enhancing students’ resilience (Fang et al., 2025), while adolescents can reduce depressive symptoms and increase life satisfaction through greater participation in extracurricular activities (Wang et al., 2023). However, it is important to note that academic interventions improve academic outcomes but do not substantially reduce academic anxiety (Fishstrom et al., 2025). Adolescents with low participation levels may experience greater social isolation and academic difficulties at school, leading to increased psychological stress (Wentzel, 2022). Therefore, the most effective approach is to promote high-frequency, high-quality parent–child interactions and leverage the positive effects of a highly adaptive school model: High-frequency, high-quality parent–child interactions enhance adolescents’ emotional security, emotional regulation abilities, and self-efficacy. Adolescents in school settings are more likely to actively engage in social and academic activities. Through highly adaptive school approaches, they continually gain social recognition and a sense of self-achievement, forming a positive cycle of “family emotional support–school achievement feedback” (Izzo et al., 2022), thereby improving their overall mental health.

While the observed effect sizes in this study are in the small-to-medium range (parent–child interaction: medium effects; school adaptation: small effects), these findings retain practical significance for several reasons. First, mental health is influenced by numerous factors simultaneously, including genetic, neurobiological, social, and environmental determinants (Bronfenbrenner and Morris, 2006; Baskin-Sommers et al., 2026). Any single predictor would therefore be expected to account for only a modest portion of total variance. Second, as Funder and Ozer (2019) have argued, even small effects can have meaningful cumulative consequences when they operate consistently over time and across large populations (Funder and Ozer, 2019; Carey et al., 2023). In the context of a nationally representative sample, even modest improvements in parent–child interaction quality could translate into meaningful reductions in depressive symptoms at the population level. Third, a recent meta-analytic study suggests that typical effect sizes in psychological research are smaller than previously assumed (Götz et al., 2022), and our findings are consistent with this updated benchmark. Future research may benefit from incorporating additional mediating and moderating variables to identify the conditions under which these effects are amplified.

### 4.4 Comparison with recent related studies

In recent years, a growing body of research using CEPS data have examined the relationship between parent–child interaction and adolescent development outcomes. Parent–child relationships significantly influence adolescents’ mental health, with parental control playing a moderating role (Xu and Ren, 2025); other studies have confirmed a significant negative correlation between parent–child interaction and depressive symptoms and that depressive symptoms and negative peer relationships mediate the relationship between parent–child interaction and delinquent behavior (Wang Y. et al., 2025; Wang H. et al., 2025; Wang X. et al., 2025). Peers’ parent–child relationships have a positive impact on students’ math and language scores (Lao, 2025); classroom context studies further revealed that prosocial behavior in the classroom has a compensatory effect on children of parents who are neglectful—exerting a greater influence on their prosocial behavior—while deviant behavior in the classroom has an amplifying effect on the same group—exerting a greater influence on their deviant behavior (Xie, 2023). At the level of family functioning and educational involvement, research shows that home-school collaboration initiatives significantly enhance parental educational involvement; however, their effects vary significantly depending on family socioeconomic status (SES)—high-SES families are able to utilize institutional resources more effectively to strengthen educational involvement, while low-SES families benefit relatively less (Li, 2026). Furthermore, although family interactions and academic support do not directly influence adolescents’ deviant behavior, they exert significant indirect effects through mediating pathways, including attentional bias, negative emotions, and peer deviance (Dou et al., 2025).

The aforementioned studies have revealed the mechanisms through which parent–child interactions and school-related factors influence adolescents’ mental health and developmental outcomes from various perspectives, including the quality of parent–child relationships, peer effects, classroom contexts, and institutional support. However, few studies have combined parent–child interaction patterns and types of school adjustment within a single analytical framework to systematically examine their differential effects on multidimensional mental health indicators—depressive symptoms and subjective wellbeing. The contribution of this study lies in identifying distinct groups based on parent–child interaction and school adjustment through latent profile analysis, revealing the combined predictive effects of different combinations of interaction patterns and adjustment types on adolescents’ mental health, thereby providing empirical evidence for interventions grounded in a family-school collaboration perspective.

### 4.5 Limitations and future research

This study also has certain limitations. First, the cross-sectional data used in this study preclude establishing causal relationships between parent–child interaction patterns, school adaptation types, and adolescent mental health. Savitz and Wellenius (2023) suggest that cross-sectional studies are often viewed “pejoratively” and are considered incapable of providing useful information for causal inference (Savitz and Wellenius, 2023). Further research indicates that cross-sectional designs can be used to explain associations but cannot support inferences regarding causality, directionality, or temporality (Egea-Zerolo et al., 2026). Accordingly, future longitudinal tracking studies are needed to further validate causal mechanisms. Second, the classification of school adaptation styles in the cluster analysis relied solely on the dimensions of active versus sedentary lifestyles, failing to adequately consider other crucial factors such as academic adaptation (Li H. et al., 2024; Li L. et al., 2024), peer relationships (Lao, 2025b), and teacher-student relationships (Ye and Wang, 2024). Furthermore, methodological studies indicate that individual-centered approaches, such as latent profile analysis (LPA), also face challenges in achieving correct classification (Cintron et al., 2023). This limitation may affect the comprehensiveness and accuracy of the classification. Finally, although this study uses the latest national survey data, their timeliness requires ongoing monitoring and updating. Additionally, the effect sizes of some variables are relatively small, and potential influencing factors not included in the study—such as socioeconomic status and peer relationships—may exist. Research shows that socioeconomic status (Luo et al., 2025) is an important predictor of adolescents’ mental health, and peer relationships (Xie, 2023) have also been shown to have a direct and significant impact on adolescents’ mental health. Future research could increase the sample size, incorporate additional influencing factors, and delve deeper into the interactions among these factors to more comprehensively reveal the mechanisms affecting adolescent mental health.

## 5 Conclusion

Based on the large-scale national CEPS sample, this study examined the independent and combined effects of parent–child interaction patterns and types of school adjustment on adolescents’ mental health (depressive symptoms and subjective wellbeing). Focusing on four hypotheses, the results are as follows: Hypothesis 1 (parent–child interaction and school adjustment are significantly correlated with mental health indicators) was fully supported; the directions of the correlations were consistent with theoretical expectations. Hypothesis 2 (different patterns of parent–child interaction have a significant association with adolescents’ mental health) was fully supported: Significant differences in mental health levels were found across interaction patterns, with the low-frequency group exhibiting the most adverse outcomes. Hypothesis 3 (there are significant differences in mental health levels among adolescents with different types of school adjustment) was fully supported: The detached adaptation group showed the poorest mental health indicators. Hypothesis 4 (parent–child interaction patterns and school adjustment types have a significant combined association with adolescents’ mental health) was supported: Both variables demonstrated significant incremental predictive power for depression (*ΔR^2^* = 0.064) and wellbeing (*ΔR^2^* = 0.087). Effect sizes ranged from small to moderate, consistent with the multifactorial and complex nature of socio-ecological factors’ influence on mental health. In summary, parent–child interaction and school adjustment are important factors to consider in interventions for adolescents’ mental health, providing empirical evidence for mental health promotion practices from a family-school collaboration perspective.

## Data Availability

Publicly available datasets were analyzed in this study. This data can be found at: Data from the China Education Panel Survey (CEPS), funded by the China Survey and Data Center at Renmin University of China (中国教育追踪调查 http://ceps.ruc.edu.cn/).
