# Correction: A study on the correlations between parent–child interaction, school adaptation type, and adolescent mental health

**DOI:** 10.3389/fpsyg.2026.2007584

**Published:** 2026-10-15

**Authors:** Xinqian Yin, Yan Wang, Yashan Qu, Lanlan Luo

**Affiliations:** 1 School of Marxism, Guilin Medical University, Guilin, China; 2 School of Education, Guangxi Normal University, Guilin, China; 3 Key Research Base of Humanities and Social Sciences in Guangxi Universities Guangxi Higher Education Development Research Center, Guilin, China; 4 School of Humanities and Management, Guilin Medical University, Guilin, China; 5 School of Educational Sciences, Guangdong Polytechnic Normal University, Guangzhou, China

**Keywords:** adolescents, depressive symptoms, happiness index, mental health, parent–child interaction, school adjustment

Affiliation “School of Humanities and Management, Guilin Medical University, Guilin, China” was omitted from the paper. This affiliation has now been added for author Yashan Qu.

Affiliation “School of Educational Sciences, Guangdong Polytechnic Normal University, Guangzhou, China” was erroneously given as “School of Educational Sciences, Guangdong Technical Normal University, Guangzhou, China”.

The original version of this article has been updated.

